# Supplementary figures and images for: Regulation of the heat stress response in Arabidopsis by MPK6-targeted phosphorylation of the heat stress factor HsfA2
Source: PeerJ. 2013 Apr 2;1:e59. doi: 10.7717/peerj.59 (PMC3628891; doi:10.7717/peerj.59)

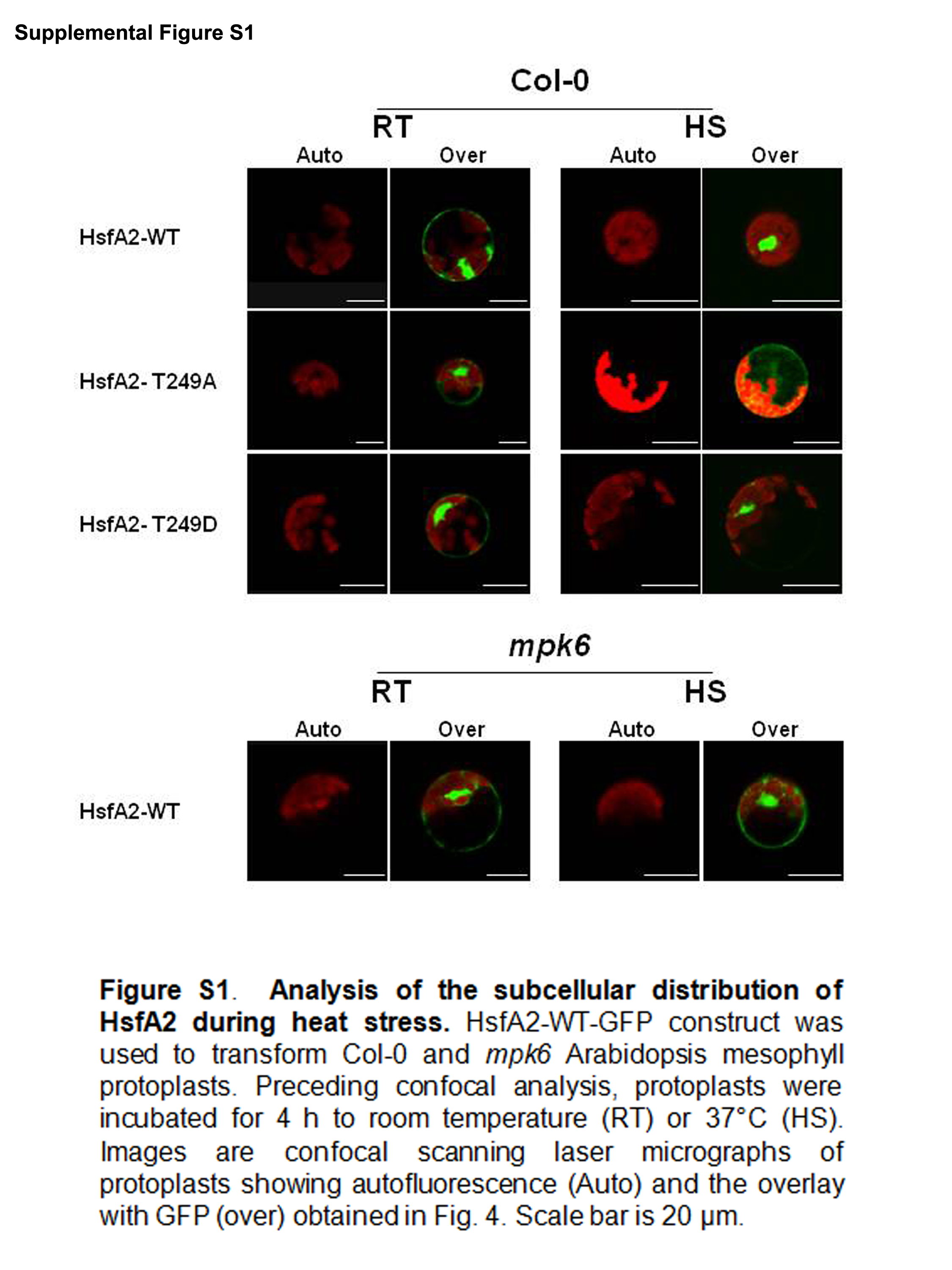

Supplement: Supplemental Figure S1 [file peerj-01-59-s001.png]

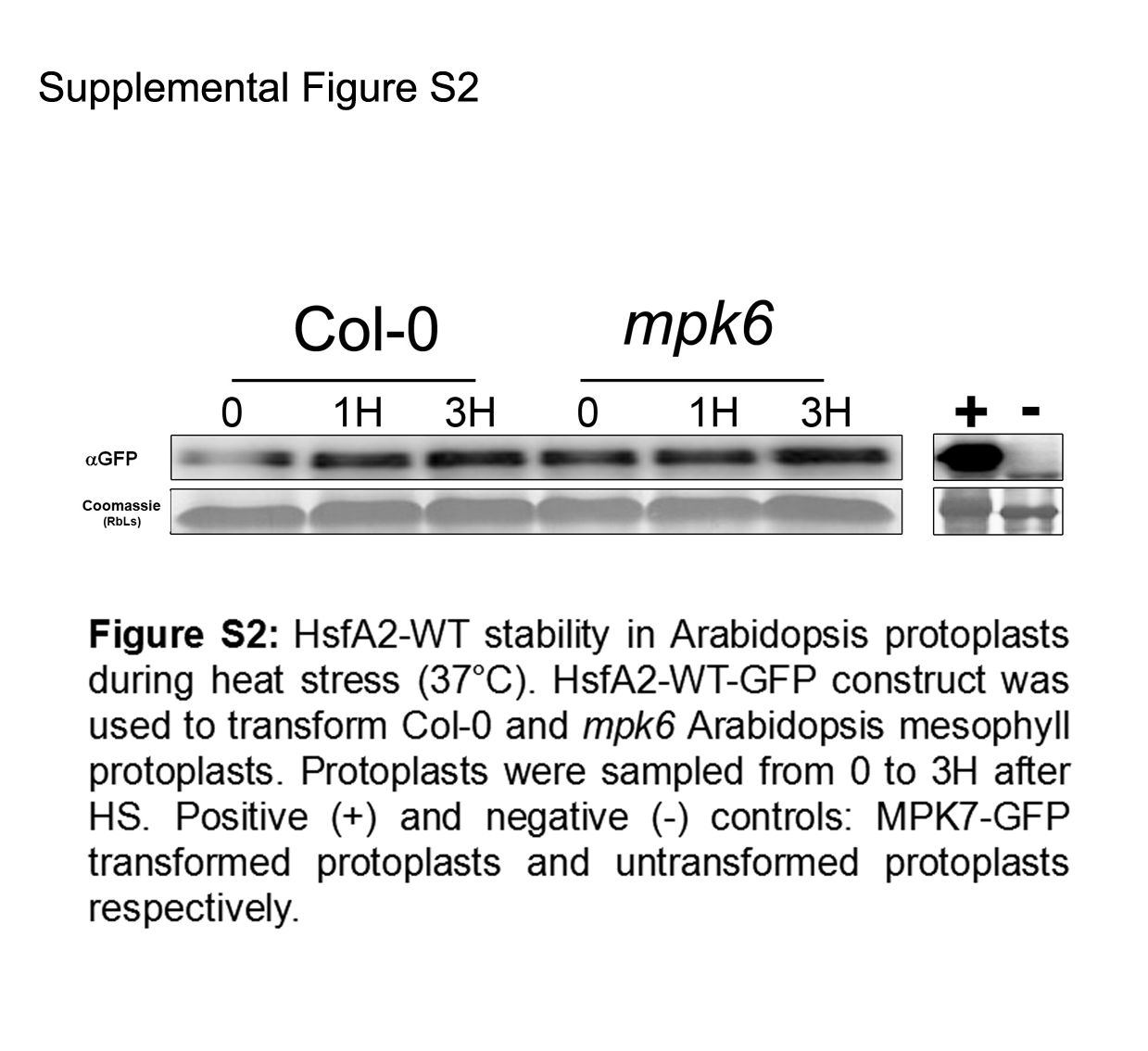

Supplement: Supplemental Figure S2 [file peerj-01-59-s002.png]

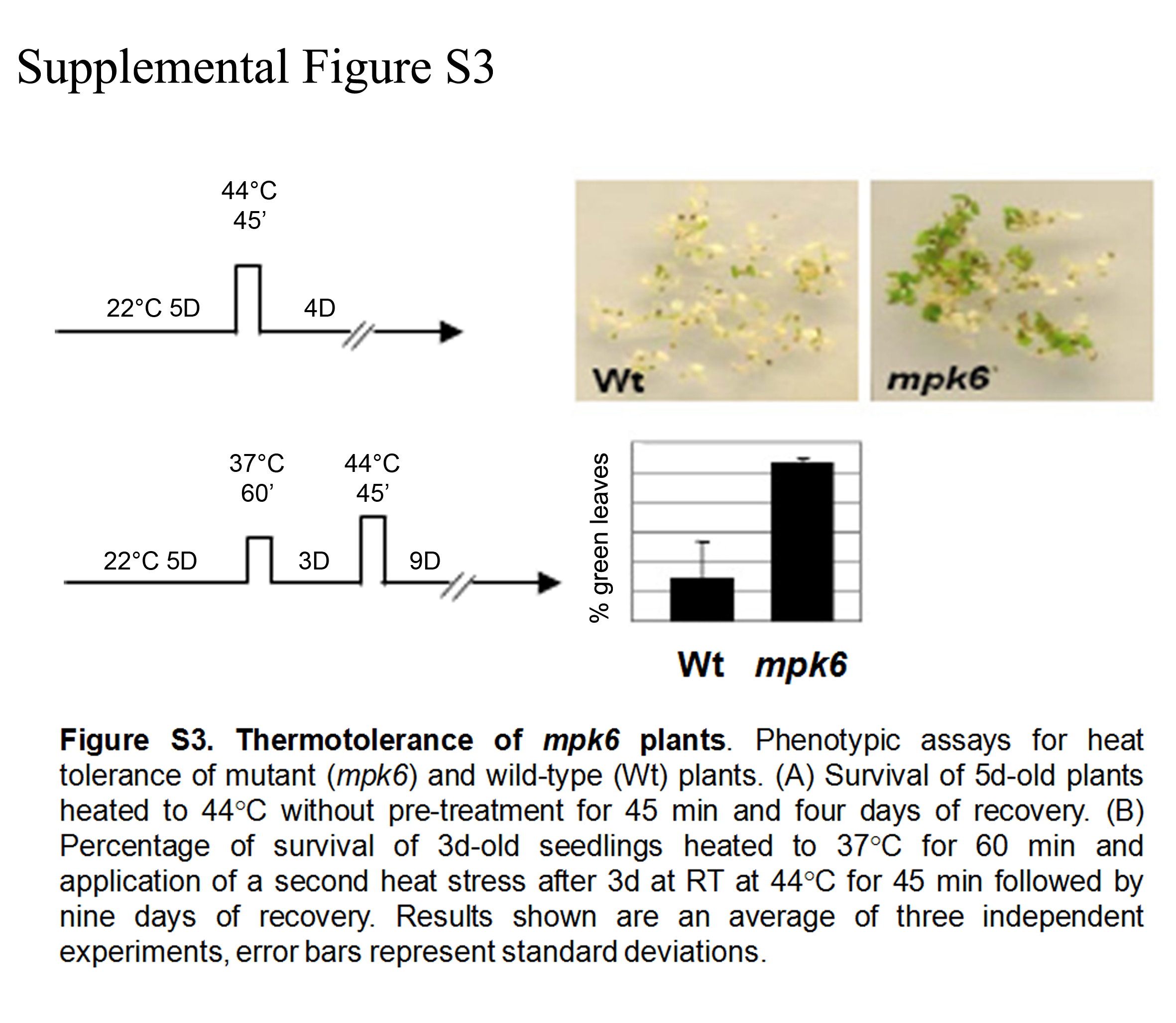

Supplement: Supplemental Figure S3 [file peerj-01-59-s003.png]
