# Supplementary material for: Regulation of the heat stress response in Arabidopsis by MPK6-targeted phosphorylation of the heat stress factor HsfA2
Source: PeerJ. 2013 Apr 2;1:e59. doi: 10.7717/peerj.59 (PMC3628891; doi:10.7717/peerj.59)
Supplement: Supplemental Table S1 [file peerj-01-59-s004.pdf]

Supplemental table S1

Arabidopsis thaliana Hsf Sequences used in Fig 2A (Name of the gene/ATG number). Cter sequences are starting just after the RR-K/R/N motif (underlined in the sequence) ending the Dna Binding Domain of Hsf transcriptions factors as described in Konak et al., 2004.

HsfA1a|At4g17750

MFVNFKYFSFFIRTKMDGVTGGGTNIGEAVTAPPPRNPHPATLLNANSLPPPFLSKTYDMVEDPATDAIVS
WSPTNNSFIVWDPPEFSRDLLPKYFKHNNFSSFVRQLNTYGFRKVDPDRWEFANEGFLRGQKHLLKKISRR
KSVQGHGSSSSNPQSQQLSQGQGSMAALSSCVEVGKFGLEEEVEQLKRDKNVLMQELVKLRQQQQTTDN
KLQVLVKHLQVMEQRQQQIMSFLAKAVQNPTFLSQFIQKQTDSNMHVTEANKKRRLREDSTAATESNSHS
HSLEASDGQIVKYQPLRNDSMMWNMMKTDDKYPFLDGFSSPNQVSGVTLQEVLPITSGQSQAYASVPSGQ
PLSYLPSTSTSLPDTIMPETSQIPQLTRESINDFPTENFMDTEKNVPEAFISPSPFLDGGSVPIQLEGIPEDPEIDE
LMSNFEFLEEYMPESPVFGDATTLNNNNNNNNNNNNNNNNNNNNTNGRHMDKLIEELGLLTSETEH

HsfA1b|At5g16820

MESVPESVPSPNSNTPSIPPPVNSVPPFLSKTYDMVDDPLTNEVVSWSSGNNSFVVWSAPEFSKVLLPKYFK
HNNFSSFVRQLNTYGFRKVDPDRWEFANEGFLRGRKQLLKSIVRRKPSHVQQNQQQTQVQSSSVGACVEV
GKFGIEEEVERLKRDKNVLMQELVRLRQQQQATENQLQNVGQKVQVMEQRQQQMMSFLAKAVQSPGFL
NQLVQQNNNDGNRQIPGSNKKRRLPVDEQENRGDNVANGLNRQIVRYQPSINEAAQNMLRQFLNTSTSPR
YESVSNNPDSFLLGDVPSSTSVDNGNPSSRVSGVTLAEFSPNTVQSATNQVPEASLAHHPQAGLVQPNIGQS
PAQGAAPADSWSPEFDLVGCETDSGECFDPIMAVLDESEGDAISPEGEGKMNELLEGVPKLPGIQDPFWEQ
FFSVELPAIADTDDILSGSVENNDLVLEQEPNEWTRNEQQMKYLTEQMGLLSSEAQRK

HsfA1d|At1g32330

MDVSKVTTSDGGGDSMETKPSPQPQPAAILSSNAPPPFLSKTYDMVDDHNTDSIVSWSANNNSFIVWKPPE
FARDLLPKNFKHNNFSSFVRQLNTYGFRKVDPDRWEFANEGFLRGQKHLLQSITRRKPAHGQGQGHQRSQ
HSNGQNSSVSACVEVGKFGLEEEVERLKRDKNVLMQELVRLRQQQQSTDNQLQTMVQRLQGMENRQQQ
LMSFLAKAVQSPHFLSQFLQQQNQQNESNRRISDTSKKRRFKRDGIVRNNDSATPDGQIVKYQPPMHEQAK
AMFKQLMKMEPYKTGDDGFLLGNGTSTTEGTEMETSSNQVSGITLKEMPTASEIQSSSPIETTPENVSAASE
ATENCIPSPDDLTLPDFTHMLPENNSEKPPESFMEPNLGGSSPLLDPDLLIDDSLSFDIDDFPMDSDIDPVDYG
LLERLLMSSPVPDNMDSTPVDNETEQEQNGWDKTKHMDNLTQQMGLLSPETLDLSR

HsfA1e|At3g02990

MGTVCESVATAKSSTAVMSSIPPFLSKTYDMVDDPLTDDVVSWSSGNNSFVVWNVPEFAKQFLPKYFKHN
NFSSFVRQLNTYGFRKVDPDRWEFANEGFLRGQKQILKSIVRRKPAQVQPPQQPQVQHSSVGACVEVGKF
GLEEEVERLQRDKNVLMQELVRLRQQQQVTEHHLQNVGQKVHVMEQRQQQMMSFLAKAVQSPGFLNQ
FSQQSNEANQHISESNKKRRLPVEDQMNSGSHGVNGLSRQIVRYQSSMNDATNTMLQQIQQMSNAPSHES
LSSNNGSFLLGDVPNSNISDNGSSSNGSPEVTLADVSSIPAGFYPAMKYHEPCETNQVMETNLPFSQGDLLP
PTQGAAASGSSSSDLVGCETDNGECLDPIMAVLDGALELEADTLNELLPEVQDSFWEQFIGESPVIGETDEL
ISGSVENELILEQLELQSTLSNVWSKNQQMNHLTEQMGLLTSDALRK

HsfA2|At2g26150

MEELKVEMEEETVTFTGSVAASSSVGSSSSPRPMEGLNETGPPPFLTKTYEMVEDPATDTVVSWSNGRNSF
VVWDSHKFSTTLLPRYFKHSNFSSFIRQLNTYGFRKIDPDRWEFANEGFLAGQKHLLKNIKRRRNMGLQNV
NQQGSGMSCVEVGQYGFDGEVERLKRDHGVLVAEVVRLRQQQHSSKSQVAAMEQRLLVTEKRQQQMM
TFLAKALNNPNFVQQFAVMSKEKKSLFGLDVGRKRRLTSTPSLGTMEENLLHDQEFDRMKDDMEMLFAA
AIDDEANNSMPTKEEQCLEAMNVMMRDGNLEAALDVKVEDLVGSPLDWDSQDLHDMVDQMGFLGSEP

HsfA4c|At5g45710
MDENNGGSSSLPPFLTKTYEMVDDSSSDSVVAWSENNKSFIVKNPAEFSRDLLPRFFKHKNFSSFIRQLNTY
GFRKVDPEKWEFLNDDFVRGRPYLMKNIHRRKPVHSHSLVNLQAQNPLTESERRSMEDQIERLKNEKEGL
LAELQNQEQERKEFELQVTTLKDRLQHMEQHQKSIVAYVSQVLGKPGLSLNLENHERRKRRFQENSLPPSS
SHIEQVEKLESSLTFWENLVSESCEKSGLQSSSMDHDAAESSLSIGDTRPKSSKIDMNSEPPVTVTAPAPKTG
VNDDFWEQCLTENPGSTEQQEVQSERRDVGNDNNGNKIGNQRTYWWNSGNVNNITEKAS

HsfA5|At4g13980
MNGALGNSSASVSGGEGAGGPAPFLVKTYEMVDDSSTDQIVSWSANNNSFIVWNHAEFSRLLLPTYFKHN
NFSSFIRQLNTYGFRKIDPERWEFLNDDFIKDQKHLLKNIHRRKPIHSHSHPPASSTDQERAVLQEQMDKLS
REKAAIEAKLLKFKQQKVVAKHQFEEMTEHVDDMENRQKKLLNFLETAIRNPTFVKNFGKKVEQLDISAY
NKKRRLPEVEQSKPPSEDSHLDNSSGSSRRESGNIFHQNFSNKLRLELSPADSDMNMVSHSIQSSNEEGASP
KGILSGGDPNTTLTKREGLPFAPEALELADTGTCPRRLLLNDNTRVETLQQRLTSSEETDGSFSCHLNLTLAS
APLPDKTASQIAKTTLKSQELNFNSIETSASEKNRGRQEIAVGGSQANAAPPARVNDVFWEQFLTERPGSSD
NEEASSTYRGNPYEEQEEKRNGSMMLRNTKNIEQLTL

HsfA6a|At5g43840
MDYNLPIPLEGLKETPPTAFLTKTYNIVEDSSTNNIVSWSRDNNSFIVWEPETFALICLPRCFKHNNFSSFVR
QLNTYGFKKIDTERWEFANEHFLKGERHLLKNIKRRKTSSQTQTQSLEGEIHELRRDRMALEVELVRLRRK
QESVKTYLHLMEEKLKVTEVKQEMMMNFLLKKIKKPSFLQSLRKRNLQGIKNREQKQEVISSHGVEDNGK
FVKAEPEEYGDDIDDQCGGVFDYGDELHIASMEHQGQGEDEIEMDSEGIWKGFVLSEEEMCDLVEHFI

HsfA7a|At3g51910
MMNPFLPEGCDPPPPPQPMEGLHENAPPPFLTKTFEMVDDPNTDHIVSWNRGGTSFVVWDLHSFSTILLPR
HFKHSNFSSFIRQLNTYGFRKIEAERWEFANEEFLLGQRQLLKNIKRRNPFTPSSSPSHDACNELRREKQVL
MMEIVSLRQQQQTTKSYIKAMEQRIEGTERKQRQMMSFLARAMQSPSFLHQLLKQRDKKIKELEDNESAK
RKRGSSSMSELEVLALEMQGHGKQRNMLEEEDHQLVVERELDDGFWEELLSDESLASTS

HsfA8|At1g67970
MVKSTDGGGGSSSSSSVAPFLRKCYDMVDDSTTDSIISWSPSADNSFVILDTTVFSVQLLPKYFKHSNFSSFI
RQLNIYGFRKVDADRWEFANDGFVRGQKDLLKNVIRRKNVQSSEQSKHESTSTTYAQEKSGLWKEVDILK
GDKQVLAQELIKVRQYQEVTDTKMLHLEDRVQGMEESQQEMLSFLVMVMKNPSLLVQLLQPKEKNTWR
KAGEGAKIVEEVTDEGESNSYGLPLVTYQPPSDNNGTAKSNSNDVNDFLRNADMLKFCLDENHVPLIIPDL
YDDGAWEKLLLLSPSRKKTKKQENIVKKGKDDLTLEEEEEDGTMELDKSYMLKLISEEMEKPDDFEFGQL
TPERSRNLEILTEQMELLASNE

HsfA9|At5g54070
MTAIPNVVDIESSSSSLCQETATETVTVERGSSDSSSKPDDVVLLIKEEEDDAVNLSLGFWKLHEIGLITPFLR
KTFEIVDDKVTDPVVSWSPTRKSFIIWDSYEFSENLLPKYFKHKNFSSFIRQLNSYGFKKVDSDRWEFANEG
FQGGKKHLLKNIKRRSKNTKCCNKEASTTTTETEVESLKEEQSPMRLEMLKLKQQQEESQHQMVTVQEKI
HGVDTEQQHMLSFFAKLAKDQRFVERLVKKRKMKIQRELEAAEFVKKLKLLQDQETQKNLLDVEREFMA
MAATEHNPEPDILVNNQSGNTRCQLNSEDLLVDGGSMDVNGRIEIE

HsfB1|At4g36990
MTAVTAAQRSVPAPFLSKTYQLVDDHSTDDVVSWNEEGTAFVVWKTAEFAKDLLPQYFKHNNFSSFIRQL
NTYGFRKTVPDKWEFANDYFRRGGEDLLTDIRRRKSVIASTAGKCVVVGSPSESNSGGGDDHGSSSTSSPG
SSKNPGSVENMVADLSGENEKLKRENNNLSSELAAAKKQRDELVTFLTGHLKVRPEQIDKMIKGGKFKPV
ESDEESECEGCDGGGGAEEGVGEGLKLFGVWLKGERKKRDRDEKNYVVSGSRMTEIKNVDFHAPLWKSS
KVCN

HsfB2b|At4g11660
MPGEQTGETPTVAGVGGGGAGCSAGNSGGSSGCGAGGGGGGSGGGGGGGGDSQRSIPTPFLTKTYQLVE
DPVYDELISWNEDGTTFIVWRPAEFARDLLPKYFKHNNFSSFVRQLNTYGFRKVVPDRWEFSNDCFKRGE
KILLRDIQRRKISQPAMAAAAAAAAAAAVAASAVTVAAVPVVAHIVSPSNSGEEQVISSNSSPAAAAAAIGG
VVGGGSLQRTTSCTTAPELVEENERLRKDNERLRKEMTKLKGLYANIYTLMANFTPGQEDCAHLLPEGKP
LDLLPERQEMSEAIMASEIETGIGLKLGEDLTPRLFGVSIGVKRARREEELGAAEEEDDDRREAAAQEGEQS
SDVKAEPMEENNSGNHNGSWLELGK

HsfB3|At2g41690
MEDAGEHLRCNDNVNDEERLPLEFMIGNSTSTAELQPPPPFLVKTYKVVEDPTTDGVISWNEYGTGFVVW
QPAEFARDLLPTLFKHCNFSSFVRQLNTYGFRKVTTIRWEFSNEMFRKGQRELMSNIRRRKSQHWSHNKSN
HQVVPTTTMVNQEGHQRIGIDHHHEDQQSSATSSSFVYTALLDENKCLKNENELLSCELGKTKKKCKQLM
ELVERYRGEDEDATDESDDEEDEGLKLFGVKLE

HsfB4|At1g46264
MAMMVENSYGGYGGGGGERIQLMVEGQGKAVPAPFLTKTYQLVDDPATDHVVSWGDDDTTFVVWRPP
EFARDLLPNYFKHNNFSSFVRQLNTYGFRKIVPDRWEFANEFFKRGEKHLLCEIHRRKTSQMIPQQHSPFMS
HHHAPPQIPFSGGSFFPLPPPRVTTPEEDHYWCDDSPPSRPRVIPQQIDTAAQVTALSEDNERLRRSNTVLMS
ELAHMKKLYNDIIYFVQNHVKPVAPSNNSSYLSSFLQKQQQQQPPTLDYYNTATVNATNLNALNSSPPTSQ
SSITVLEDDHTNHHDQSNMRKTKLFGVSLPSSKKRSHHFSDQTSKTRLVLDQSDLALNLMTASTR
