# Supplementary material for: Regulation of the heat stress response in Arabidopsis by MPK6-targeted phosphorylation of the heat stress factor HsfA2
Source: PeerJ. 2013 Apr 2;1:e59. doi: 10.7717/peerj.59 (PMC3628891; doi:10.7717/peerj.59)
Supplement: Supplemental Table S2 [file peerj-01-59-s005.pdf]

Supplemental table S2

Primers used to generate HsfA2-WT-GFP, HsfA2-T249A-GFP, HsfA2-T249D-GFP and MPK7-GFP constructs in the p2GWF7 plasmid.

Hsfa2-T249D-F CGGAGGCTTACTTCTGATCCAAGCTTGGGGAC
Hsfa2-T249D-R GTCCCCAAGCTTGGATCAGAAGTAAGCCTCCG
Hsfa2-T249A-F CGGAGGCTTACTTCTGCTCCAAGCTTGGGGAC
Hsfa2-T249A-R GTCCCCAAGCTTGGAACAGAAGTAAGCCTCCG
Hsfa2NS-GW2-CterF
GGGGACAAGTTTGTACAAAAAAGCAGGCTCATGGAAGAACTGAAAGTGGAAATGGAG
Hsfa2NS-GW2-R
GGGGACCACTTTGTACAAGAAAGCTGGGTCAGGTTCCGAACCAAGAAAACCCA
MPK7NS-GW2-CterF
GGGGACAAGTTTGTACAAAAAAGCAGGCTCATGGCGATGTTAGTTGAGCCACC
MPK7NS-GW2-R
GGGGACCACTTTGTACAAGAAAGCTGGGTTGGGTTTTCTTGGTTCGGAACCT
